# Atypical Pyoderma Gangrenosum Mimicking an Infectious Process

**DOI:** 10.1155/2014/589632

**Published:** 2014-06-12

**Authors:** Derek To, Aaron Wong, Valentina Montessori

**Affiliations:** ^1^Faculty of Medicine, University of British Columbia, Vancouver, BC, Canada V6Z 1Y6; ^2^Department of Dermatology and Skin Science, University of British Columbia, Vancouver, BC, Canada V6Z 1Y6; ^3^Division of Infectious Diseases, Department of Medicine, University of British Columbia, Vancouver, BC, Canada V6Z 1Y6

## Abstract

We present a patient with atypical pyoderma gangrenosum (APG), which involved the patient's arm and hand. Hemorrhagic bullae and progressive ulcerations were initially thought to be secondary to an infectious process, but a biopsy revealed PG. Awareness of APG by infectious disease services may prevent unnecessary use of broad-spectrum antibiotics.

## 1. Case

We present a 48-year-old, hepatitis C positive man with a previous history of intravenous drug use. In 2013, his hepatitis C level was found to be 855697 IU/mL of genotype 3a, but the patient denied using any treatment for this condition. In addition, he had a fifteen-year history of rheumatoid arthritis and a four-year history of recurrent episodes of nodular lesions on the arms that would ulcerate. He was admitted to hospital for suspicion of cellulitis of his right hand with extensive lymphangitis and suppurative lymphadenitis in the right axilla.

Ten years prior to presenting, the patient had suffered a gunshot wound in his right lower calf. It was treated conservatively with wound care. The wound was chronically infected and treated with various antibiotics, though never completely resolving. Six years later he began developing a new nodular lesion at this site, which resolved with antibiotics and wound dressings. However three years later at the same wound site, he developed a blistering lesion, which ruptured into an ulcer after it was struck by a bicycle pedal. He presented to the emergency department and cultures from the wound grew* Staphylococcus aureus*. No evidence of cellulitis or lymphangitis was noted on examination. Over the following five days, the suppurative lesion continued to progress in size and did not respond to cephalexin. An incision and drainage was performed with wound cultures growing* Streptococcus constellatus* and* Enterobacter cloacae*. Ciprofloxacin and penicillin were then started. A negative pressure dressing was applied with subsequent wound care. Eventually, a skin graft was performed several months afterwards.

Concurrently, the patient was also developing tender nodular lesions on his extensor surfaces of his forearms bilaterally, which were thought to be rheumatoid nodules. The patient recalled that these recurrent nodules would blister and then rupture, resulting in a slow-healing ulcer. Furthermore, he has repeatedly presented to the emergency department in the past for daily intravenous antibiotic treatment of his wounds. He became colonized with methicillin resistant* Staphylococcus aureus* (MRSA). The patient reported that he had previously taken hydroxychloroquine for his rheumatoid arthritis. While he was on the hydroxychloroquine, he noticed marked improvement of his lesions but discontinued this medication as he felt his rheumatoid arthritis was not active.

During the patient's most recent admission, he presented with a two-week history of multiple tender bullous lesions on the flexural surface of the right forearm and dorsum of his right hand, one of which he intentionally ruptured with drainage of pus. Another bullous lesion on the hand was lanced in the emergency department, and the patient was started on daily intravenous cefazolin and vancomycin. Over the following several days, the patient developed fever and chills with the lanced bullae developing into enlarging tender ulcers. He was admitted, and his antibiotic coverage was broadened to piperacillin/tazobactam and vancomycin.

**Figure 1:**
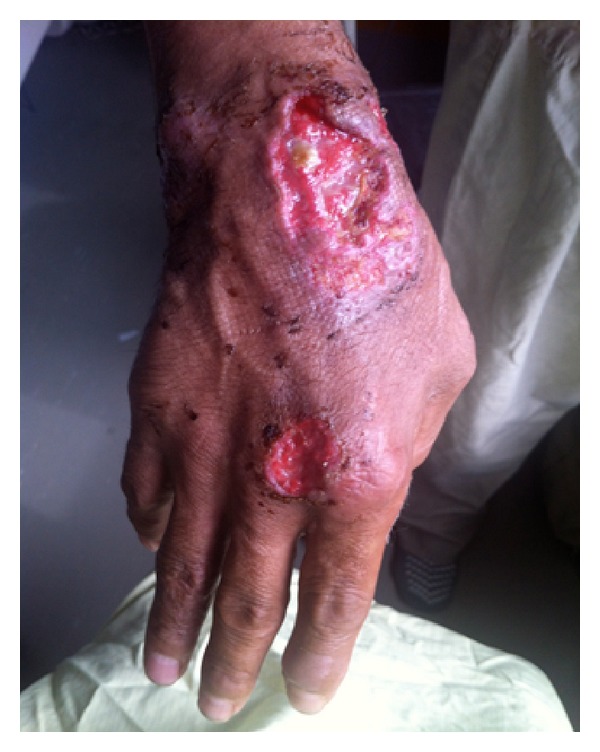
Ruptured bullae after being lanced two days earlier on the dorsum of the right hand.

**Figure 2:**
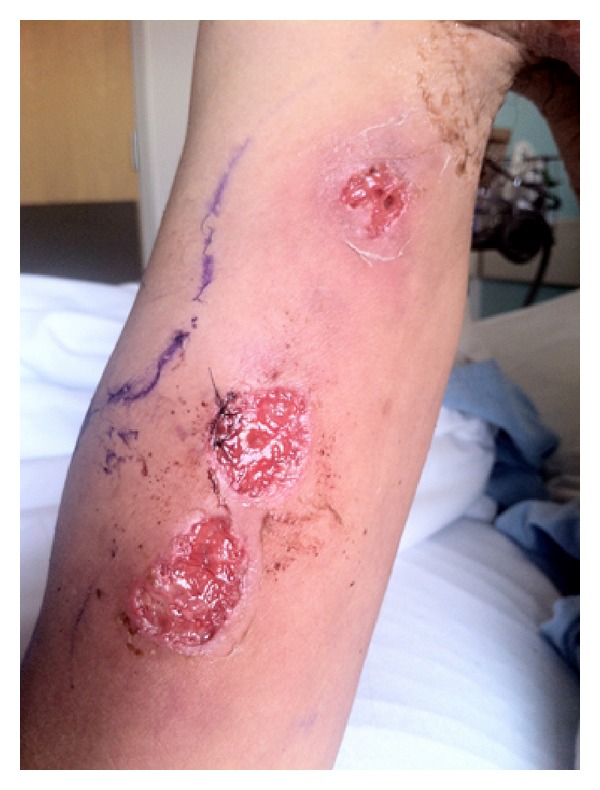
Right medial bicep showing three well demarcated ulcerated lesions with a beefy cribriform erythematous base.

On examination, the patient had a large right axillary collection with spontaneous drainage. This collection seemed to track anteriorly to the border of his pectoralis muscle accompanied by tenderness and increased warmth. Two previously lanced ulcerated lesions with well demarcated borders and a mild amount of surrounding erythema were found on the dorsum of the right hand with several tender nodules sitting along the streaking lymphangitis (Figure 1), which extended from his wrist to the medial aspect of his right upper arm (Figure 2).

A wound culture from the right hand showed +3 polymorphonuclear leukocytes on Gram stain and then grew group A streptococcus and diphtheroids. A CT chest scan displayed multiple tiny abscesses in the pectoralis major.

Over the next several days, the patient's wounds were stabilized in size and two punch biopsies from the right arm ulcers were performed. One biopsy was for routine hematoxylin and eosin staining (H&E) and the other for culture. The biopsy of the ulcer showed a neutrophilic infiltrate consistent with pyoderma gangrenosum and wound cultures were negative for atypical mycobacteria and deep fungal infection. Subsequently, the patient was discharged home with outpatient follow-up of his rheumatoid arthritis and pyoderma gangrenosum.

## 2. Discussion

Pyoderma gangrenosum (PG) is an uncommon inflammatory ulcerative dermatosis with an unknown etiology. First described by Brocq in 1916 and with further characterization by Perry and Brunsting [1], PG is classified into four types: classic (ulcerative), bullous, pustular, and vegetative [2]. The lesions typically develop over the lower limbs in patients during their fourth or fifth decade of life. Classic cutaneous findings include a tender, violaceous, scalloped, undermined ulcer edge and a red, friable base. Prior to ulcer formation, the preceding lesion is usually an erythematous to violaceous papule or papulopustule. Typically, pyoderma gangrenosum expands rapidly and is extremely painful. Diagnosis is established by excluding other causes for cutaneous ulcerations through clinical history, wound culture, and biopsy [2]. A clinical feature in 30% of PG cases includes the phenomenon of pathergy, in which minor trauma, including venipuncture, results in the formation of pyoderma gangrenosum at that site [3]. Variations in PG exist as Bennett et al. described atypical PG (APG) as having lesions typically occurring on the upper extremities [4]. These lesions usually begin as tense hemorrhagic bullae, which ulcerate and heal without the typical cribriform scarring [4]. 50% of PG cases are idiopathic, while the other 50% of cases are associated with a systemic disease. APG is most commonly associated with hematologic diseases and malignancies (hairy cell leukemia, myelogenous leukemia, myelofibrosis, and monoclonal gammopathy) followed by seronegative arthritis [4]. Other associations include inflammatory bowel disease, rheumatoid arthritis, and hepatitis C [5].

Unfortunately, there are no histopathologic features that are pathognomonic for PG. Generally, biopsy of the ulcer shows heavy neutrophil infiltration with evidence of hemorrhage and necrosis [6]. PG is histologically a neutrophilic dermatosis, which can mimic a variety of conditions depending on its clinical course upon presentation. Wolfe et al. described a case of APG which mimicked squamous cell Case Reports in Infectious Diseases 3 carcinoma on the hand [7], and Lee et al. reported a case of PG misdiagnosed as a diabetic foot infection [8]. Huish et al. illustrated a case series of APG being misdiagnosed as a pyogenic wound infection and treated inappropriately with antibiotics and/or surgical intervention [9]. Our case of APG initially had the hemorrhagic bullae and nodules diagnosed as rheumatoid nodules. Upon rupture of these bullae on the hand and upper arm, a cellulitic wound infection was diagnosed as lymphangitis of the axilla. The significant wounds of PG are often secondarily infected with bacteria, making management even more of a challenge.

No gold standard algorithm exists for management of pyoderma gangrenosum. Local wound management, treating the underlying condition, and pain and infection control are the mainstays in management of pyoderma gangrenosum. Wound management needs to be tailored for the type of lesions present. For more exudative type lesions, absorptive dressings can be used such as hydrocolloids, whereas chronic less exudative lesions may require moisture retentive occlusion dressings [10]. For widespread lesions, systemic therapy has traditionally been prednisone; however, infliximab, a TNF-alpha inhibitor, is emerging as a first line treatment [10]. Adjunctive treatment includes topical medications such as tacrolimus [10] and intralesional corticosteroid injections [11]. Alternative options would also include immunomodulators such as cyclosporine, mycophenolate mofetil, azathioprine, and methotrexate [11, 12]. In addition, hyperbaric oxygen therapy is a safe and cost-effective alternative therapy [13]. In more refractory cases, IVIG and alkylating agents, such as cyclophosphamide, have been used [10]. For cases where an underlying systemic disease is present and believed to be associated with PG, treating the systemic disease may prove to be beneficial. In a case report by Kondo et al., a patient with hepatitis C and PG-like lesions, which was initially refractory to immunosuppressive therapy, was initiated on pegylated interferon-*α*-2b and ribavirin therapy. Viral response was achieved, and the PG-like lesions resolved [14].

The role of antibiotics is limited as a review of the literature has shown only four cases of PG that have been responsive to minocycline, which is possibly due to its anti-inflammatory effect [15]. Sulphonamides and sulfones have also been shown to control PG in a small number of cases, but again their anti-inflammatory action is postulated to have more of an effect than their antimicrobial capabilities [16].

Currently, there is no strong evidence showing that surgery is effective as this intervention may induce pathergy [11]. However, five of the eleven cases in a literature review by Huish et al. underwent unnecessary irrigation and debridement for unrecognized PG [9]. In the case report by Huish et al., the patient initially had a progressive ulcerating wound of the index finger, which was refractory to multiple irrigation and debridement attempts and parental antibiotics. Eventually, the patient had an unnecessary amputation [9]. This patient was later diagnosed to have PG.

In our case, the presentation of APG was quite challenging to differentiate from other infectious processes. However, the poor response to antibiotics and eventual skin biopsy were clues in realizing that this was indeed underlying pyoderma gangrenosum. Awareness of this condition by inpatient and outpatient infectious disease practitioners will help avoid unnecessary broad-spectrum antibiotics and possibly more invasive interventions such as surgery.
